# Psychosocial work factors affecting mental health of young workers: a systematic review

**DOI:** 10.1007/s00420-022-01907-y

**Published:** 2022-08-17

**Authors:** Malte van Veen, Karen M. Oude Hengel, Roosmarijn M. C. Schelvis, Paulien M. Bongers, Johannes C. F. Ket, Allard J. van der Beek, Cécile R. L. Boot

**Affiliations:** 1 Netherlands Organisation for Applied Scientific Research TNO, Unit Healthy Living, Leiden, The Netherlands; 2 Amsterdam UMC location Vrije Universiteit Amsterdam, Public and Occupational Health, Boelelaan 1117, Amsterdam, The Netherlands; 3 Body@Work, Research Center on Work, Health and Technology, TNO/VUmc, Amsterdam, The Netherlands; 4 Amsterdam UMC, location University of Amsterdam, Public and Occupational Health, Meibergdreef 9, Amsterdam, The Netherlands; 5 Amsterdam Public Health, Societal Participation & Health, Amsterdam, The Netherlands; 6 Medical Library, Vrije Universiteit Amsterdam, Amsterdam, The Netherlands

**Keywords:** Systematic review, Psychosocial work factors, Mental health, Young workers

## Abstract

**Objective:**

For the general working population, robust evidence exists for associations between psychosocial work exposures and mental health. As this relationship is less clear for young workers, this systematic review aims at providing an overview of the evidence concerning psychosocial work factors affecting mental health of young workers.

**Methods:**

The electronic databases used were PubMed, Web of Science, and PsycINFO and were last searched in October 2021. The eligible outcomes included depression-, stress-, burnout- and anxiety-related complaints, and fatigue, excluding clinical diagnoses and suicide-related outcomes. Only studies with workers aged 35 years or younger were included, which reported at least one association between a psychosocial work factor as exposure and a mental health complaint as outcome. Studies had to be in English, German or Dutch. Risk of bias was assessed using an instrument from the National Heart, Lung, and Blood Institute. Data synthesis was conducted using GRADE.

**Results:**

In total 17 studies were included in this systematic review, including data from 35,600 young workers in total. Across these studies 86 exposure-outcome associations were reported. Nine exposure-outcome associations could be synthesised. The application of the GRADE framework led to one “low” assessment for the association between psychosocial job quality and mental health. The certainty of evidence for the other eight associations in the synthesis was very low.

**Conclusions:**

The current systematic review disclosed a high degree of uncertainty of the evidence due to conceptually fuzzy outcomes and exposures as well as large heterogeneity between studies.

**Supplementary Information:**

The online version contains supplementary material available at 10.1007/s00420-022-01907-y.

## Introduction

Adverse psychosocial working conditions are widely recognized to play an important role for workers’ mental health, which in turn has consequences for individuals, organizations, and society as a whole. For individuals and organizations these consequences include temporary or sustained sickness absence from work and lower productivity (Lerner and Henke 2008). On a societal level, the OECD has estimated that within Europe the costs of mental health complaints, both clinical and subclinical, were more than € 600 billion in 2015 (4.1% of EU GDP) (OECD and European Union 2018). Psychosocial working conditions have been found to be crucial for a worker’s mental health and improving these conditions will diminish their negative impact (Andrea et al. 2009; Stansfeld and Candy 2006).

Several established models formulate how poor psychosocial work can lead to workers’ mental health complaints (e.g., the job-demand-control model, the effort-reward-imbalance model and the organizational justice model) (Siegrist and Wahrendorf 2016). A meta-review on work-related mental health complaints, qualitatively synthesizing 37 systematic reviews lists three broad, partially overlapping, work-related risk factor categories associated with mental health complaints: (1) imbalanced job design (e.g., high job demands), (2) occupational uncertainty (e.g., high job insecurity), and (3) lack of value and respect in the workplace (e.g., workplace conflict/bullying) (Harvey et al. 2017). Other risk factors allocated to more than one category, e.g., job control as part of imbalanced job design and occupational uncertainty, and effort-reward imbalance as part of imbalanced job design and lack of value and respect in the workplace were also associated with mental health complaints (Harvey et al. 2017). Another meta-review, assessing a broad spectrum of work-related health outcomes, including mental health outcomes (Niedhammer et al. 2021), and a systematic review on stress-related disorders, only including prospective cohort studies (van der Molen et al. 2020), both have reported similar conclusions, supporting that high job demands, effort-reward imbalance, job insecurity, and low organizational justice are associated with mental health complaints. For job insecurity, van der Molen et al. (2020) only found an association for men. Evidence is mixed for job control, which shows a weaker association with mental health complaints than the other mentioned factors (van der Molen et al. 2020). Niedhammer et al. (2021) combined job demands and job control into job strain as one factor, so that the role of job control cannot be assessed individually.

The reviews above concern the general working population. However, young workers deserve particular attention. This is, firstly, because research suggests a cohort effect for today’s young adults’ mental health that might persist into later life (Twenge et al. 2019) with young people reporting increasingly worse mental health compared to older people (Hewlett et al. 2021). Secondly, being unable to work or being unable to work as much as one wants due to mental health issues in early life can turn into a lifelong disadvantage for young adults. To prevent mental health complaints early, a proper understanding of the work-related factors that affect young workers’ mental health is crucial.

The findings from the general working population cannot naturally be assumed to be applicable to young workers. Research on job satisfaction during school-to-work transition and from lifespan developmental psychology suggests that young workers systematically differ from their older colleagues in terms of work-related psychosocial needs and accompanying risks for mental health complaints. Instability around one’s work, for instance, can have more impact on younger workers than on older workers (Schmitt and Unger 2019). Additionally, young workers are exposed more often to some risk factors than older workers, such as conflicts at work and temporary working arrangements (Milner et al. 2017).

Two systematic reviews assessed the effect of psychosocial work conditions on mental health complaints of young workers (Law et al. 2020; Shields et al. 2021). Law et al. (2020) identified ten work-related risk factors that are in line with those for the general population listed by Harvey et al. (2017), except for job boredom, which Harvey et al. (2017) did not address. Law et al. (2020) did not provide an assessment of the certainty of the evidence across studies. Shields et al. (2021) concluded that some low-certainty evidence exists for an association of low job control, sexual harassment, and low psychosocial job quality with mental health complaints of young workers.

The current systematic review builds on the two aforementioned earlier reviews by applying a broader conceptualization of mental health complaints, including burnout and related concepts such as mental fatigue. Regarding the exposures, particularly factors that might affect young workers, such as fear of missing out, role stress, and social support at work, are included in the search strategy. In contrast to the two previous reviews, that defined young workers as not older than 30 years, the current systematic review defines young workers as individuals who are 35 years or younger. The extension of the age criterion for this review can be considered appropriate, because a growing share of the population follows longer education trajectories, leading to a later entry into the labour market as reflected by a recent OECD definition of young adults as those being between 25 and 34 years old (OECD 2020). Thus, the current study includes a broader scope on both the exposure and outcome. Hence, this systematic review provides not only an updated, but also a more complete picture of the state of the literature including a more systematic assessment of the certainty of the evidence by applying the GRADE approach (Huguet et al. 2013).

This systematic review aims at providing an overview of the evidence concerning psychosocial work factors affecting mental health of young workers.

## Methods

This systematic is reported according to the PRISMA statement (Page et al. 2021) and the review protocol was submitted beforehand to PROSPERO (PROSPERO ID CRD42021259886).

### Search strategy and study selection

Titles and abstracts were retrieved from the databases PubMed, Clarivate Analytics/Web of Science Core Collection, and Ebsco/APA PsycINFO up to and including October 7th, 2021 by MVV and JCFK, using search terms related to (1) young workers, (2) psychosocial factors, (3) mental health, and (4) study design. The full search strategy is provided in supplementary file 1.

Regarding the population, only studies with workers aged 35 years or younger were included. Regarding the exposures and outcomes, studies were included if they reported an association between a psychosocial work factor as exposure and a mental health complaint as outcome. The eligible outcomes include depression-, stress-, burnout-, exhaustion- and anxiety-related complaints, as well as fatigue, excluding clinical diagnoses and suicide-related outcomes. Intervention studies and qualitative studies were excluded. Studies had to be in English, German or Dutch.

Two reviewers (MVV and KOH) independently assessed titles and abstracts for eligibility using Rayyan (Ouzzani et al. 2016). If consensus on eligibility could not be reached, then a third author (CB) was asked as tie-breaker. Subsequently, two reviewers (MVV and KOH) independently assessed the full text of the selected articles. Authors of potentially eligible studies were contacted when maximum age was not explicitly reported in the article. Again, if consensus on inclusion could not be reached, a third author (CB) was consulted.

In addition to the primary search, a complimentary citation search based on the included studies was conducted. This was done backwards by one author (MVV) by screening the reference list of the included studies and forwards by using Google Scholar to find studies that cited the included studies.

### Risk of bias assessment

Risk of bias assessment per study was conducted independently by two researchers (MVV and KOH) using the items from the Quality Assessment Tool for Observational Cohort and Cross-Sectional Studies from the National Heart, Lung, and Blood Institute (National Heart, Lung, and Blood Institute 2019). Risk of bias items were: clear statement of research questions; specification of study population; participation rate above 50%; sample size justification; measuring of exposure prior to outcome; sufficient timeframe for seeing effect; examination of different levels of exposure; measurement of exposure clearly defined, and valid, reliable, consistently implemented across all study participants; repeated measures of exposure; measurement of outcome clearly defined, and valid, reliable, and consistently implemented across all study participants; statistical adjustment of potential key confounding variables; and: overall risk of bias assessment per study. For the current systematic review, gender and education were considered key confounders that an analysis had to include in order to get a no risk of bias judgement on the respective item. Following the tool’s guidelines, the overall risk of bias assessment was not mechanically determined but determined using the overall judgement of the authors based on all items.

### Data extraction

Data from the included studies was extracted by one author (MVV) using a pre-piloted form that was developed for this systematic review. The extracted data items were: authors; year of publication; sample origin country; sample size; occupational information; age range; outcome; outcome measurement; exposure; exposure measurement; type of analysis; included control variables; and statistical coefficient to describe the exposure-outcome association. Whenever confounder-adjusted coefficients were available, those were extracted. Three authors (KOH, CB, and AvdB) checked two studies each for optimizing the data extraction.

### Data synthesis and certainty assessment

A quantitative synthesis of the data was not planned due to the expected inhomogeneity of outcomes and exposures. All decisions concerning harmonization of terminology (hereafter referred to as harmonization) were made after data extraction. For a tabulated overview, all exposure-outcome associations are sorted by outcome. For further synthesis, conceptually equivalent exposures were harmonized and data was synthesized using the GRADE framework (Huguet et al. 2013).

Within the GRADE framework each exposure-outcome association starts with an initial quality level of evidence judgement. Based on nine items this initial level can be downgraded or upgraded. The level of evidence is downgraded when individual studies show biases (based on study-level risk of bias assessment), estimates are imprecise (based on confidence intervals), evidence is inconsistent, exposures or outcomes are measured indirectly, and when publication bias is likely for the particular association. The level of evidence is upgraded when there is evidence for a dose–response relationship, when the effect size is substantial, and when confounding is unlikely to affect the overall association. All studies found for this review were observational studies. The initial level of evidence for observational studies is “low quality of evidence”, indicating that “our confidence in the effect estimate is limited: the true effect may be substantially different from the estimate of the effect” (Huguet et al. 2013).

## Results

**Fig. 1:**
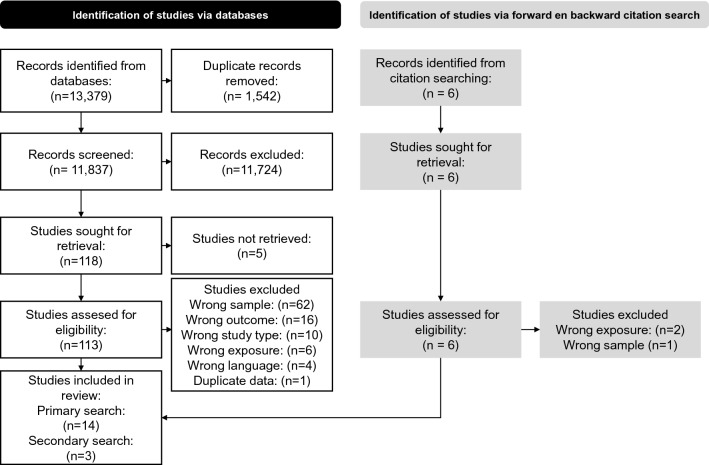
Flow diagram of literature search

The flow of studies into the review is shown in Fig. 1. The full texts of 113 original studies were assessed in our primary search after having screened 11,837 deduplicated titles and abstracts. Five studies selected for full text reading based on title and abstract could not be retrieved. Finally, the primary search resulted in inclusion of 14 studies (Akkermans et al. 2009, 2013a; Berth et al. 2003; Cheng et al. 2013; Haley et al. 2013; Klug 2020; Lachmann et al. 2020; Lee et al. 2015; Raspe et al. 2020; Salmela-Aro and Upadyaya 2018; Shi et al. 2018; Wiesner et al. 2005; Zimmerman et al. 2004; Zoer et al. 2011). Of the 99 excluded studies, 62 studies did not fit the study population criteria, 16 studies did not report an eligible outcome, ten studies did not fit the design criteria, and six studies did not report an eligible exposure. Four studies were in a non-eligible language and one study contained data duplicate with another study. Citation searching led to additional inclusion of three studies (Akkermans et al. 2013b; Elovainio et al. 2007; Milner et al. 2017), adding up the total count of included studies to 17 for the current systematic review.

### Study characteristics

Table 1 shows a detailed description of the characteristics of the 17 included studies. Within seven studies the study population had a maximum age of 35 years, six studies took 30 years as the maximum age, and for four other studies the maximum age was 25 (Akkermans et al. 2009), 28 (Wiesner et al. 2005), 31 (Elovainio et al. 2007), and 33 (Berth et al. 2003) years. For eight studies, participants were sampled from particular occupational domains (manufacturing, transport, finance, education, combination of network services, administration, and chemistry, and three times healthcare). In the other nine studies young workers from the general working population participated. Sixteen studies used questionnaires to obtain exposure data and one study (Zimmerman et al. 2004) used a job exposure matrix for exposure measurement. Across all 17 studies, 14 different outcomes and 59 different exposures were reported leading to 86 exposure-outcome associations. Three studies had a longitudinal design with Akkermans et al. (2013a) measuring the exposure prior to the outcome to be analysed with a structural equation model, and Milner et al. (2017) and Klug (2020) applying a longitudinal fixed-effects analysis to estimate within effects. The other 14 studies applied cross-sectional designs. All studies combined included 35,600 young workers.

**Table 1:** Study characteristics of the included studies

First author (year), sample origin Country	Study design	Sample	Age in years	Outcome	Outcome measurement	Exposure	Exposure measurement^a,b^
Akkermans et al. (2009), the Netherlands	Cross-sectional	2535 workers from Netherlands working condition survey 2007	18–25	Emotional exhaustion	Based on utrecht burnout scale	Autonomy	Based on JCQ
						Cognitive demands	Self-constructed scale
						Emotional demands	Based on COPSOQ
						Social support from supervisor	Based on JCQ
						Social support from colleagues	Based on JCQ
						Task variation	Based on JCQ
						Workload	Based on JCQ
Akkermans et al. (2013a), the Netherlands	2 waves longitudinal; exposure on wave 1, outcome wave 2	1284 workers, wave 1 from Netherlands working condition survey 2008, wave 2 follow-up	18–30	Emotional exhaustion	Based on utrecht burnout scale	Job resources	Latent factor based on autonomy, supervisor support, colleague support (based on JCQ)
						Job demand	Latent factor based on work pressure, emotional workload, physical workload (based on JCQ)
Akkermans et al. (2013b), the Netherlands	Cross-sectional	305 workers from a dutch educational institution and a dutch multinational	16–30	Emotional exhaustion	Subscale from utrecht burnout scale	Job demands	Latent factor based on work pressure, physical demands, and emotional demands
						Job resources	Latent factor based on social support, autonomy, and opportunities for development
Berth et al. (2003), Germany	Cross-sectional	411 workers from a sample that was representative for the GDR at study begin	28–33	Anxiety	HADS (hospital anxiety and depression scale—German version)	Job insecurity	Self-constructed scale
				Depression	HADS (hospital Anxiety and Depression scale—German version)	Job insecurity	Self-constructed scale
				Fatigue	Subscale from GBB-24 (Gießen symptom checklist)	Job insecurity	Self-constructed scale
				Psychological distress	Subscale from SCL-9 (symptom check list)	Job insecurity	Self-constructed scale
Cheng et al. (2013), Taiwan	Cross-sectional	4892 workers randomly sampled from general work population	20–29	Burnout	Personal burnout subscale of Chinese version of copenhagen burnout inventory	Job insecurity	Self-constructed scale
						Job control	Chinese version of JCQ
						Workplace justice	Validated Scale
						Psychological job demands	Based on Chinese version of JCQ
Elovainio et al. (2007), Finland	Cross-sectional	3873 workers from birth cohort	31	Psychological distress	SCL-25	Job control	Self-constructed scale
						Job demands	Self-constructed scale
						Job strain	Composite measure based on job control and job demands; high job strain = high demand and low control
Haley et al. (2013), South Africa	Cross-sectional	134 junior managers financial sector	18–30	Exhaustion (subscale of burnout)	Self-constructed burnout scale	Colleague support	Self-constructed scale
						Emotional load	Self-constructed scale
						Job pressure	Self-constructed scale
						Job information	Self-constructed scale
						Mental load	Self-constructed scale
						Participation in decision making	Self-constructed scale
						Role clarity	Self-constructed scale
						Supervisor support	Self-constructed scale
Klug (2020), Germany	Longitudinal	963 young workers from representative household panel study	18–30	Mental health	Mental health component scale from SF-12	Subjective job insecurity	Self-constructed scale
Lachmann et al. (2020), Germany	Cross-sectional	390 doctors	< = 35	Burnout-risk	COPSOQ	Effort-reward-imbalance	ERI
						Effort	Subscale from ERI
						Reward	Subscale from ERI
Lee et al. (2015), South Korea	Cross-sectional	1141 female workers at manufacturing plant	18–35	Anxiety symptoms	Korean version of beck anxiety inventory	Interpersonal conflict	KOSS-SF
						Insufficient job control	KOSS-SF
						Job demand	KOSS-SF
						Job insecurity	KOSS-SF
						Lack of reward	KOSS-SF
						Occupational climate	KOSS-SF
						Organizational system	KOSS-SF
Milner et al. (2017), Australia^c^	Longitudinal	9723 workers from the representative HILDA study	< = 30	Mental health	Mental component summary of SF-36	Psychosocial job quality (trichotomized based on number of adverse adversities)	Validated scale
Raspe et al. (2020), Germany	Cross-sectional	1060 doctors and nurses	< = 35	Burnout	Burnout scale from COPSOQ	Career mobility	Subscale from ERI-16
						Collaboration	Work situation questionnaire for physicians (FAÄ) and for nurses (FAP)
						Effort	Subscale from ERI-16
						Effort-reward-imbalance	ERI16—subscales aggregated in 1 ratio
						Experienced aggression	Self-constructed scale
						Job security	Subscale from ERI-16
						Recognition	Subscale from ERI-16
Salmela-Aro and Upadyaya (2018), Finland	Cross-sectional	263 workers from three large organizations	< 35	Work burnout	Bergen burnout inventory	Authoritarian management	Self-constructed scale
						ICT demands	Self-constructed scale
						Interpersonal work demands	Self-constructed scale
						Multicultural demands	Self-constructed scale
						Role in the organization	Self-constructed scale
						Team climate	Self-constructed scale
Shi et al. (2018), China	Cross-sectional	696 novice nurses (< 3 service years)	19–35	Anxiety	Scale based on previous research (Abdel-Khalek 2006)	Workplace incivility	Self-constructed scale
				Burnout	Chinese version of the maslach burnout inventory-general survey	Workplace incivility	Self-constructed scale
Wiesner et al. (2005), United States	Cross-sectional	583 workers earlier graduating from suburban New York high schools	21–28	Depressive symptoms	Center for epidemiological studies—depression scale (CES-D)	High cognitive demands	Adapted from Hurrell and McLaney (1988)
						High job boredom	Scale from Frone and McFarlin (1989)
						High workload	Adapted from Quinn and Staines (1979)
						Low autonomy	Adapted from Quinn and Staines (1979)
						Low skill variety	Adapted from Cammann et al. (1983)
Zimmerman et al. (2004), United States	For outcome one wave of longitudinal cohort is used, for exposure job factors were taken from O*Net based on job codes	7278 workers from a cohort that was nationally representative at cohort begin	27–35	Depressive symptoms	Center for epidemiological studies—depression scale (CES-D)	Job security	Scores from occupations profiles taken from O*Net database
						Moral	Scores from occupations profiles taken from O*Net database
						Opposition	Scores from occupations profiles taken from O*Net database
						Physical discomfort	scores from occupations profiles taken from O*Net database
						Recognition	scores from occupations profiles taken from O*Net database
						Sociability	scores from occupations profiles taken from O*Net database
Zoer et al. (2011), the Netherlands	Cross-sectional	69 workers from railway company	22–35	Burnout	Utrecht burnout scale	Emotional load	From Van Veldhoven et al. (2002)
						Lack of social support from colleagues	From Van Veldhoven et al. (2002)
						Lack of social support from supervisor	From Van Veldhoven et al. (2002)
						Low work autonomy	From Van Veldhoven et al. (2002)
						Mental load	From Van Veldhoven et al. (2002)
						Work pressure	From Van Veldhoven et al. (2002)
				Stress complaints	Distress subscale from dutch version of 4DSQ	Emotional load	From Van Veldhoven et al. (2002)
						Lack of social support from colleagues	From Van Veldhoven et al. (2002)
						Lack of social support from supervisor	From Van Veldhoven et al. (2002)
						Low work autonomy	From Van Veldhoven et al. (2002)
						Mental load	From Van Veldhoven et al. (2002)
						Work pressure	From Van Veldhoven et al. (2002)
				Work-related fatigue	Scale for need for recovery after working time	Emotional load	From Van Veldhoven et al. (2002)
						Lack of social support from colleagues	From Van Veldhoven et al. (2002)
						Lack of social support from supervisor	From Van Veldhoven et al. (2002)
						Low work autonomy	From Van Veldhoven et al. (2002)
						Mental load	From Van Veldhoven et al. (2002)
						Work pressure	From Van Veldhoven et al. (2002)

^a^Self-constructed scale: Scale is not reported to be based on previous research

^b^*JCQ* job content questionnaire, *COPSOQ* copenhagen psychosocial Questionnaire, *KOSS-SF* korean occupational stress scale (short form), *ERI* effort reward imbalance scale, *ERI (16)* ERI short form

^c^Data are taken from supplementary material in which the analysis are reported for workers only, excluding students and unemployed

### Risk of bias assessment

Table 2 shows a risk of bias assessment. Two studies reported a repeated measurement of the exposure. Eight of the 17 studies took potential confounding by education level and gender into account. Eleven studies were rated as poor, four as fair and two studies as good.

**Table 2:** Risk of Bias Assessment per study using the National Heart, Lung, and Blood Institute framework

First author (year)	Research questions clearly stated?	Specified study population?	Participation rate of 50% of higher?	Justification of the sample size?	Exposure measured prior to outcome?	Sufficient timeframe to see an effect?	Exposure measurement	Outcome measurement	Appropriate dealt with confounders?	Overall rating: good/fair/poor^d^
Akkermans et al. (2009)	Yes	Yes	Unclear	No	No	No	Yes	Yes	Yes	Fair
Akkermans et al. (2013a)	Yes	Yes	Unclear	No	Yes	Yes	Yes	Yes	Yes	Fair
Akkermans et al. (2013b)	Yes	Yes	Yes	No	No	No	Yes	Yes	No	Poor
Berth et al. (2003)	No	Yes	Yes	No	No	No	No	Yes	No	Poor
Cheng et al. (2013)	Yes	Yes	Yes	No	No	No	Yes	Yes	No	Poor
Elovainio et al. (2007)	Yes	Yes	Yes	No	No	No	Yes	Yes	No	Poor
Haley et al. (2013)	Yes	Yes	Unclear	No	No	No	No	No	No	Poor
Klug (2020)	Yes	Yes	Yes	No	Yes	Yes	Yes	Yes	Yes	Good
Lachmann et al. (2020)	Yes	Yes	No	No	No	No	Yes	Yes	Yes	Poor
Lee et al. (2015)	Yes	Yes	Yes	No	No	No	Yes	Yes	No	Poor
Milner et al. (2017)	Yes	Yes	Yes^e^	No	Yes	Yes	Yes	Yes	Yes	Good
Raspe et al. (2020)	Yes	Yes	No	No	No	No	Yes	Yes	No	Poor
Salmela-Aro and Upadyaya (2018)	Yes	Yes	No	No	No	No	No	Yes	No	Poor
Shi et al. (2018)	Yes	Yes	Yes	No	No	No	Yes	Yes	Yes	Fair
Wiesner et al. (2005)	Yes	Yes	Yes	No	No	No	Yes	Yes	Yes	Fair
Zimmerman et al. (2004)	Yes	Yes	Yes	No	No	No	No	Yes	Yes	Poor
Zoer et al. (2011)	Yes	Yes	No	No	No	No	Yes	Yes	No	Poor

^d^Following the tool’s guidelines, the overall risk of bias assessment was not mechanically determined, but determined using on the overall judgement of the authors based on all items

^e^Retention from wave to wave is > 90%, but over time this might have ended up in an overall attrition of > 50%. Overall attrition is not provided in the article

### Harmonization of exposures and outcomes for data synthesis

Exposures and outcomes that were conceptually equivalent were given the same term for a more comprehensible overview and data synthesis. The decisions on what constitutes conceptual equivalence in the context of the current systematic review was consensual and based on the experience and domain knowledge of the authors.

Concerning the exposures this applies to interpersonal conflict (including workplace incivility, experienced aggression, interpersonal work demands), rewards (including recognition), job control (including autonomy, work autonomy), job demands (including psychological job demands, work pressure, workload, job demands, job pressure), emotional demands (including emotional load), and cognitive demands (including mental load). For all other exposures the original terms were used.

The same was done for outcomes that were conceptually equivalent: anxiety symptoms (Lee et al. 2015) were harmonized as anxiety; work burnout (Salmela-Aro and Upadyaya 2018), burnout-risk (Lachmann et al. 2020), exhaustion as burnout-subscale (Haley et al. 2013), and emotional exhaustion (Akkermans et al. 2009, 2013a, b), were harmonized as burnout; depressive symptoms (Wiesner et al. 2005; Zimmerman et al. 2004) were harmonized as depression; Work-related fatigue (Zoer et al. 2011) and fatigue (Berth et al. 2003), were harmonized as fatigue. Stress complaints (Zoer et al. 2011) and psychological distress (Berth et al. 2003; Elovainio et al. 2007) were harmonized as stress.

In Table 1 the original terms are used for outcomes and exposures, whereas the harmonized terms are used in Tables 3, 4. This harmonization of terminology reduced the number of outcomes from 14 to 6, the number of exposures from 59 to 44.

**Table 3:** Associations between work-related exposures and mental health complaints

Exposure	Study	Exposure levels	Association coefficient (incl. *p* value or 95% confidence interval)^f^
*Anxiety*			
Job insecurity	Berth (2003)^g^	4-point-scale, 4 is most insecurity	***F*(3408) = 10.21; *p* < 0.001** **4 > 1; 4 > 2; 3 > 1; 3 > 2**
	Lee (2015)^h^	Trichotomized; reference: low risk/least insecurity	OR Moderate: 1.54 [0.99:2.4] **OR high: 4.52 [2.86:7.13]**
Interpersonal conflict	Lee (2015)^h^	Trichotomized; reference: low risk/least conflict	OR moderate: 1.18 [0.75:1.86] **OR high: 2.26 [1.55:3.3]**
	Shi (2018)^i^	Continuous, higher = more conflict	***β* = 0.364; *p* < 0.01**
Insufficient job control	Lee (2015)^h^	Trichotomized; reference: low risk/most control	Moderate: no observations OR high: 1.05 [0.75:1.47]
Job demand	Lee (2015)^h^	Trichotomized; reference: low risk/least demand	Moderate: no observations **OR high: 3.19 [2.27:4.49]**
Lack of reward	Lee (2015)^h^	Trichotomized; reference: low risk/most reward	**OR moderate: 1.65 [1.01:2.69]** **OR high: 2.75 [1.86:4.08]**
Occupational climate	Lee (2015)^h^	Trichotomized; reference: low risk/most supportive climate	**OR moderate: 2.53 [1.67:3.85]** **OR high: 4.52 [2.9:7.04]**
Organizational system	Lee (2015)^h^	Trichotomized; reference: low risk/most supportive system	**OR: moderate: 1.61 [1.01:2.58]** **OR high: 2.32 [1.58:3.4]**
*Burnout*			
Job demands	Akkermans (2009)^m^	Continuous, higher = more demands	Low education: ***B* = 0.55; *p* < 0.01** Intermediate education: ***B* = 0.43; *p* < 0.01** High education: ***B* = 0.43;* p* < 0.01**
	Akkermans (2013a)^n^	Continuous, higher = more demands	Low education: **Path coeff.: 0.14** High education: **Path coeff.: 0.26**
	Akkermans (2013b)^o^	Continuous, higher = more demands	**Path coefficient: 0.28**
	Cheng (2013)^t^	Trichotomized; reference: low risk/least demands	Male: OR moderate: 1.3 [0.8:1.9] **OR high: 3.2 [2.1:4.8]** Female: OR moderate: 1.3 [0.9:1.8] **OR high: 3.7 [2.6:5.2]**
	Haley (2013)^p^	Continuous, higher = more demands	***β* = 0.24; *p* = 0.03**
	Zoer (2011)^n^	Trichotomized; reference: low risk/least demands	OR moderate: 5.2 [0.5:50.2] **OR high: 17.2 [1.2:242.3]**
Cognitive demands	Akkermans (2009)^m^	Continuous, higher = more demands	Low education: *B* = − 0.05 Intermediate education: *B* = − 0.06 High education: *B* = − 0.03
	Haley (2013)^p^	Continuous, higher = more demands	*β* = − 0.03; *p* = 0.79
	Zoer (2011)^n^	Trichotomized; reference: low risk/least demands	OR moderate: 4.7 [0.2:98.8] OR high: 0.5 [0:14.6]
Colleague support	Akkermans (2009)^m^	Continuous, higher = more support	Low education: ***B* = − 0.39; *p* < 0.01** Intermediate education: ***B* = − 0.16; *p* < 0.01** High education: *B* = **− **0.13
	Haley (2013)^p^	Continuous, higher = more support	*β* = − 0.14; *p* = 0.12
	Zoer (2011)^v^	Trichotomized; reference: low risk/high support	OR moderate: 0.1 [0:3.7] OR high: 6.9 [0.4:128.8]
Emotional demands	Akkermans (2009)^m^	Continuous, higher = more demands	Low education: ***B* = 0.5; *p* < 0.01** Intermediate education: ***B* = 0.46; *p* < 0.01** High education: ***B* = 0.5; *p* < 0.01**
	Haley (2013)^p^	Continuous, higher = more demands	***β* = 0.33; *p* < 0.01**
	Zoer (2011)^v^	Trichotomized; reference: low risk/least demands	OR moderate: 19.9 [0.9:452.7] **OR high: 33.9 [1.7:678.6]**
Job control	Akkermans (2009)^m^	Continuous, higher = more control	Low education: ***B* = − 0.13; *p* < 0.01** Intermediate education: ***B* = − 0.29; *p* < 0.01** High education: *B* = 0.03
	Cheng (2013)^t^	Trichotomized; reference: low risk/most control	Male: OR moderate: 0.9 [0.6:1.2] OR high: 0.5 [0.4:0.8] Female: OR moderate: 1.2 [0.8:1.6] OR high: 1.1 [0.8:1.6]
	Zoer (2011)^v^	Trichotomized; reference: low risk/most control	OR moderate: 0.2 [0:2.4] OR high: 1 [0:22]
Interpersonal conflict	Raspe (2020)^u^	Continuous, higher = more conflict	***B* = 2.1 [0.33:3.81]**
	Salmela-Aro (2018)^w^	Continuous, higher = more conflict	**Path coefficient: 0.21**
	Shi (2018)^i^	Continuous, higher = more conflict	***β* = 0.24; *p* < 0.01**
Supervisor support	Akkermans (2009)^m^	Continuous, higher = more support	Low education: ***B* = − 0.3; *p* < 0.01** Intermediate education: ***B* = − 0.35; *p* < 0.01** High education: ***B* = **− **0.33; *p* < 0.01**
	Haley (2013)^p^	Continuous, higher = more support	*β* = 0.04; *p* = 0.75
	Zoer (2011)^v^	Trichotomized; reference: low risk/high support	OR moderate: 0.2 [0:3.4] OR high: 2.8 [0.2:51.6]
Effort	Lachmann (2020)^x^	Dichotomized; reference: low risk/least effort	**OR: 1.04 [1.02:1.05]**
	Raspe (2020)^u^	Continuous, higher = more effort	***B* = 0.8 [0.22:1.35]**
Effort-reward-imbalance	Lachmann (2020)^x^	Dichotomized; reference: low risk/beneficial balance	**OR: 7.022 [3.139:15.709]**
	Raspe (2020)^u^	Continuous, higher = more imbalance	***B* = 8.8 [6.57–11.12]**
Reward	Lachmann (2020)^x^	Dichotomized; reference: low risk/most reward	**OR: 0.96 [0.93:0.99]**
	Raspe (2020)^u^	Continuous, higher = more reward	***B* = **− **1.5 [**− **2.22:**− **0.8]**
Job resources	Akkermans (2013a)^n^	Continuous, higher = more resources	Low education: **path coeff.: **− **0.18** High education: **path coeff.: **− **0.16**
	Akkermans (2013b)^o^	Continuous, higher = more resources	**Path coefficient: **− **0.13**
Job security	Cheng (2013)^t^	Dichotomous Item; reference: low risk/most security	Male: OR: 1 [0.7: 1.3] Female: OR: 0.9 [0.7:1.1]
	Raspe (2020)^u^	Continuous, higher = more security	*B* = − 0.8 [− 0.15:0.02]
Authoritarian management	Salmela-Aro (2018)^w^	Continuous, higher = more authoritarian	**Path coefficient: 0.21**
Career mobility	Raspe (2020)^u^	Continuous, higher = more mobility	***B* = **− **0.8 [**− **1.34:**− **0.19]**
Collaboration	Raspe (2020)^u^	Continuous, higher = more collaboration	***B* = **− **1.2 [**− **1.97:**− **0.44]**
ICT demands	Salmela-Aro (2018)^w^	Continuous, higher = more demands	**Path coefficient: 0.13**
Job information	Haley (2013)^p^	Continuous, higher = more information	*β* = 0; *p* = 0.99
Participation in decision making	Haley (2013)^p^	Continuous, higher = more participation	*β* = − 0.18; *p* = 0.1
Multicultural demands	Salmela-Aro (2018)^w^	Continuous, higher = more demands	Path coefficient: 0
Role clarity	Haley (2013)^p^	Continuous, higher = more clarity	*β* = − 0.09; *p* = 0.43
Role in the organization	Salmela-Aro (2018)^w^	Continuous, higher = higher position in hierarchy	Path coefficient: 0
Task variation	Akkermans (2009)^m^	Continuous, higher = more variation	Low education: ***B* = − 0.1;** ***p* < 0.05** Intermediate education: *B* = − 0.01 High education: *B* = 0.08
Team climate	Salmela-Aro (2018)^w^	Continuous, higher = more beneficial work-relationships	Path coefficient: 0
Workplace justice	Cheng (2013)^t^	Trichotomized; reference: low risk/high justice	Male: **OR moderate: 1.6 [1:2.4]** **OR low: 5.5 [3.7:8.2]** Female: OR moderate: 1.2 [0.8:1.8] **OR low: 4.8 [3.4:6.8]**
*Depression*			
Job insecurity (Berth)/job security (Zimmerman)	Berth (2003)^g^	4-point-scale, 4 is most insecurity	***F*(3408) = 17.91; *p* < 0.001** **4 > 1; 4 > 2; 3 > 1; 3 > 2**
	Zimmerman (2004)^j^	Continuous, higher = more security	Male: SR^k^: 0.992 [0.943:1.044] Female: 1.018 [0.968:1.069]
Cognitive demands	Wiesner (2005)^l^	Continuous, higher = more demands	*β* = −0.04
Job boredom	Wiesner (2005)^l^	Continuous, higher = more boredom	***β* = 0.13; *p* < 0.001**
Low skill variety	Wiesner (2005)^l^	Continuous, higher = less variety	*β* = **0.11; *p* < 0.01**
Low job control	Wiesner (2005)^l^	Continuous, higher = less control	***β* = 0.08; *p* < 0.05**
Moral (involvement in situations that are morally difficult)	Zimmerman (2004)^j^	Continuous, higher = more moral difficulties	Male: SR: 1.026 [0.974:1.08] Female: SR: 0.995 [0.932:1.062]
Opposition (opposition to others)	Zimmerman (2004)^j^	Continuous, higher = more opposition	Male: **SR: 1.075 [1.01:1.145]** Female: SR: 1.004 [0.94:1.072]
Physically uncomfortable	Zimmerman (2004)^j^	Continuous, higher = more discomfort	Male: SR: 0.97 [0.926:1.017**]** Female: **SR: 1.07 [1.014:1.129]**
Recognition (social status of the job)	Zimmerman (2004)^j^	Continuous, higher = more recognition	Male: **SR: 0.897 [0.822:0.977]** Female: 0.994 [0.926:1.065]
Sociability (opportunities for social interaction at work)	Zimmerman (2004)^j^	Continuous, higher = more social interaction	Male: SR: 1.033 [0.974:1.095] Female: SR: 0.971 [0.927:1.017]
Workload	Wiesner (2005)^l^	Continuous, higher = more workload	*β* = 0.03
*Fatigue*			
Job demands	Zoer (2011)^v^	Trichotomized; reference: low risk/least demands	OR moderate: 5.1 [0.9:30.4] **OR high: 17.8 [2.1:149.7]**
Cognitive demands	Zoer (2011)^v^	Trichotomized; reference: low risk/least demands	No association concluded and no statistics reported
Emotional demands	Zoer (2011)^v^	Trichotomized; reference: low risk/least demands	OR moderate: 1.7 [0.3:10.3] OR high: 5.2 [0.9:30]
Low supervisor support (Zoer)	Zoer (2011)^v^	Trichotomized; reference: low risk/high support	OR moderate: 0.2 [0:1.6] OR high:1.5 [0.3:8.3]
Low colleague support (Zoer)	Zoer (2011)^v^	Trichotomized; reference: low risk/high support	OR moderate: 0.2 [0:1.6] OR high: 0.6 [0.1:4.8]
Low job control (Zoer)	Zoer (2011)^v^	Trichotomized; reference: low risk/most control	No association concluded and no statistics reported
Job insecurity	Berth (2003)^g^	4-point-scale, 4 is most insecurity	***F*(3408) = 5.7; *p* < 0.01** **4 > 1; 4 > 2; 3 > 1**
*Mental health*			
Job insecurity	Klug (2020)^q^	Continuous, higher = more insecurity	β = 0.06
Psychosocial job quality	Milner (2017)^r^	4 groups based on number of psychosocial job adversities	**0 adversities: *β* = 0.85 [0.42:1.27]** 1: β = 0.4 [**− **0.26:0.53] **2: *β* = − 0.68 [− 1.11:− 0.25]** ** > = 3: *β* = − 1.96 [− 2.55:− 1.37]**
*Stress*			
Job demands	Zoer (2011)^v^	Trichotomized based on tertiles; reference: low risk/least demands	OR moderate: 4.3 [0.9:20.5] **OR high: 6.2 [1.2:33.8]**
	Elovainio (2007)^s^	Continuous, higher = more demands	*β* = 0.03
Job control	Elovainio (2007)^s^	Continuous, higher = more control	***β* = − 0.15; *p* < 0.001**
	Zoer (2011)^v^	Trichotomized; reference: low risk/most control	No association concluded and no statistics reported
Cognitive demands	Zoer (2011)^v^	Trichotomized; reference: low risk/least demands	OR moderate: 2 [0.5:8.4] OR high: 1.5[0.3:7.7]
Colleague support	Zoer (2011)^v^	Trichotomized; reference: low risk/most support	OR moderate: 0.5 [0.1:3.0] OR high: 1.7 [0.3:9.5]
Emotional demands	Zoer (2011)^v^	Trichotomized; reference: low risk/least demands	OR moderate: 1.1 [0.3:4.9] OR high: 1.7 [0.4:7.7]
Job insecurity	Berth (2003)^g^	4-point-scale, 4 is most insecurity	***F*(3408) = 3.49; *p* < 0.05** Scheffé Post Hoc: non
Job strain	Elovainio (2007)^s^	Continuous, higher = more strain	***β* = 0.16; *p* < 0.001**
Supervisor support	Zoer (2011)^v^	Trichotomized; reference: low risk/most support	OR moderate: 0.2 [0:1.1] OR high: 0.9 [0.2:4.6]

^f^Bold font indicates statistical significance as reported by the authors; “low”, “moderate”, high” in last column refers to risk level as specified in column “exposure groups”

^g^Berth: analysis: one-way ANOVA with Scheffé test for post hoc contrasts; Confounder: none

^h^Lee: analysis: multivariable logistic regression; Confounder: sleep quality, smoking habit, risky drinking

^i^Shi: analysis: multivariable linear regression; Confounder: age, gender, hospital level, working years, education level, department distribution

^j^Zimmerman: analysis: univariable zero-inflated negative binomial regression; Confounder: all other exposures, machine pace, physical discomfort, “wage premium”, highest grade completed, income, age, employer-provided insurance, private insurance, government insurance, being married, being divorced, being black, being Latino

^k^*SR* symptom ratio

^l^Wiesner: analysis: multivariable logistic regression, statistics reported here from model including control variables, but not other exposures; Confounder: age, gender, marital status, children, years of education, type of occupation, part-time working, labour-force experience, negative affectivity, stressful life events, work-family conflict, prior mental health problems, parental mental health problem history

^m^Akkermans (2009): analysis: multivariable linear regression; Confounder: other “demand” exposures (workload, emotional demands, cognitive demands), gender, age

^n^Akkermans (2013a): analysis: structural equation modelling; Confounder: age, gender, job change in last 12 months, partly other exposure-outcome associations in final structural equation model.

^o^Akkermans (2013b): Analysis: structural equation modelling; Confounder: non

^p^Haley: analysis: multivariable linear regression; Confounder: Other exposures

^q^Klug: analysis: longitudinal linear fixed effects regression; Confounder: age, tenure, household context, working in public sector, job change, temporary employment, interaction of education and subjective job insecurity

^r^Milner: analysis: longitudinal linear fixed effects regression; Confounder: age, highest level of education, disability/long term health condition, household structure, household income; Column “Association coefficient” indicates “Association of within-person changes in psychosocial job quality and time-varying covariates with changes in mental health”

^s^Elovainio: analysis: multivariable linear regression; Confounder: gender

^t^Cheng: analysis: multivariable logistic regression; Confounder: other exposures, working hours

^u^Raspe: analysis: multivariable regression with backward selection; Confounder: other exposures, age, gender, occupation

^v^Zoer: analysis: multivariable logistic regression; Confounder: gender, other exposures

^w^Salmela-Aro: analysis: Structural equation modeling; Confounder: personal resources, personal demands, gender, and other outcome-associations in final structural equation model

^x^Lachmann: analysis: multivariable logistic regression; Confounder: age, gender, type of work, working hours.

**Table 4:** Certainty of the evidence of using GRADE approach

Outcome	Exposure	Number of studies	Number of participants	GRADE: risk of bias within studies↓	GRADE: imprecision ↓	GRADE: inconsistency of evidence↓	GRADE: indirectness (concerning measurement and sample) ↓	GRADE: publication bias↓	GRADE dose-re-sponse relation-ship↑	GRADE: size of effect↑	GRADE: confounding↑	Overall certainty of evidence
Burnout	Cognitive demands	3(Akkermans et al. 2009; Haley et al. 2013; Cheng et al. 2013)	2738	Yes ↓	Yes ↓	Inconsistent ↓	No	Unclear	No	No	Unclear	Very low
	Colleague support	3(Akkermans et al. 2009; Haley et al. 2013; Cheng et al. 2013)	2738	Yes ↓	Yes ↓	Inconsistent ↓	No	Unclear	No	No	Unclear	Very low
	Emotional demands	3(Akkermans et al. 2009; Haley et al. 2013; Cheng et al. 2013)	2738	Yes ↓	Yes ↓	Consistent	No	Unclear	Yes↑	No	Unclear	Very low
	Interpersonal conflict	3(Raspe et al. 2020; Salmela-Aro and Upadyaya 2018; Shi et al. 2018)	2019	Yes ↓	Yes ↓	Consistent	No	Unclear	No	No	Unclear	Very low
	Job control	3(Akkermans et al. 2009; Cheng et al. 2013; Zoer et al. 2011)	7496	Yes ↓	Yes ↓	Inconsistent ↓	No	Unclear	No	No	Unclear	Very low
	Job demands	6(Akkermans et al. 2009; Akkermans 2013a, Akkermans, 2013b, Cheng et al. 2013, Haley et al. 2013; Zoer et al. 2011)	9219	Yes ↓	Yes ↓	Consistent	No	Unclear	Yes↑	No	Unclear	Very low
	Supervisor support	3(Akkermans et al. 2009; Haley et al. 2013; Cheng et al. 2013)	2738	Yes ↓	Yes ↓	Inconsistent ↓	No	Unclear	No	No	Unclear	Very low
Mental health	Psychosocial job quality	1(Milner et al. 2017)	9723	No	No	N.a	No	Unclear	Yes ↑	No	Unclear	Low^y^
	Job Insecurity	1(Klug 2020)	963	No	Yes ↓	N.a	No	Unclear	No	No	Unclear	Very low

^y^The certainty is still considered low even with no downgrade and one upgrade, because there is only one study for this exposure-outcome-association

### Overview of exposures and outcomes

Three studies measured anxiety (Berth et al. 2003; Lee et al. 2015; Shi et al. 2018). Ten studies measured burnout (Akkermans et al. 2009, 2013a, b; Cheng et al. 2013; Haley et al., 2013; Lachmann et al. 2020; Raspe et al. 2020; Salmela-Aro and Upadyaya 2018; Shi et al. 2018; Zoer et al. 2011). Three studies measured depression (Berth et al. 2003; Wiesner et al. 2005; Zimmerman et al. 2004). Two studies measured fatigue. Two studies measured mental health (Klug 2020; Milner et al. 2017), and three studies measured stress (Berth et al. 2003; Elovainio et al. 2007; Zoer et al. 2011).

Table 3 shows the results of the included studies, by displaying all 86 exposure-outcome associations sorted by the six outcomes (anxiety, burnout, depression, fatigue, mental health, stress).

### Main findings after data synthesis and certainty assessment

Table 4 shows the data synthesis. Exposure-outcome associations were included in this table when at least three studies reported a particular exposure-outcome association. Also, associations reported in the two studies with low risk of bias (Klug 2020; Milner et al. 2017) were included. This resulted in the synthesis of nine exposure-outcome associations: (1) Burnout in association with (a) cognitive demands, (b), colleague support, (c) emotional demands, (d) interpersonal conflict, (e) job control, (f) job demands, and (g) supervisor support; (2) Mental health in association with (a) psychosocial job quality and (b) job insecurity. Publication bias was very unlikely to have systematically altered the results. Across the included studies, statistically insignificant associations between exposures and outcomes were reported. An incentive to only publish significant results was unlikely to play a role, because there are no indications that authors were committed to particular theories or models.

The application of the GRADE framework led to eight certainty assessments of “very low” and one “low” assessment. The latter concerned the study by Milner et al. (2017) and the association between low psychosocial job quality and poor mental health. As the certainty of the evidence was either low or very low, the nature of the synthesized associations is not further reported.

## Discussion

This systematic review generally concludes a very low certainty of evidence on the effect of psychosocial work factors on mental health complaints of young workers. The included studies contain a myriad of exposures and outcomes as well as a substantial risk of bias. Both contributed to judgements of either very low (eight times) or low (one time) certainty in the evidence for the exposure-outcome associations.

These findings are in line with conclusions from two previous systematic reviews among young workers (Law et al. 2020; Shields et al. 2021). Both reviews concluded that the knowledge body is insufficient and called for more and better research on the topic. This conclusion is substantiated by the current review. By choosing a broad scope concerning the outcome and exposure search terms and by applying the GRADE framework, this systematic review disclosed the substantial degree of uncertainty in a more systematic way than was presented in both of the previous reviews. Nevertheless, this systematic review and the previous two reviews did find individual associations between psychosocial work factors and mental health, even though with a high degree of uncertainty. For the general population robust associations were reported between high job demands, effort-reward imbalance, job insecurity, low organizational justice and mental health complaints (Harvey et al. 2017; Niedhammer et al. 2021; van der Molen et al. 2020). It is likely that at least some of these exposures also play a role in work-related health of young workers. However, it is not clear which exposures have which effect and what the underlying mechanisms are for young workers.

Mental health is a complex phenomenon with a lack of consensus on definition and measurement. There is an ongoing debate in academia and practice about the uniqueness of the outcome constructs included in the current systematic review, e.g. discussing to which extent self-reported burnout symptoms are distinct from self-reported symptoms of depression, or whether anxiety and depression are sufficiently distinct (Kotov et al. 2021). This debate is particularly relevant for the sub-clinical populations in the current systematic review, for which symptoms are less clearly manifested. This makes systematically reviewing and synthesizing the literature challenging and becomes particularly visible in this review due to the relatively low number of studies.

While some of the included studies integrated different types of job demands into one latent construct (Akkermans et al. 2013a; b), other studies (Zoer et al. 2011) report job demands as discrete construct that exists next to other types of work-related demands. In general, studies barely provided reasonings as to why a particular exposure was chosen to be studied. Another issue is that the theoretical models behind the exposures are–despite their merits in understanding occupational mental health–justifiably described as “ways of thinking” (Siegrist and Wahrendorf 2016) that are not leading to clear and testable hypotheses when it comes to applying them, so that these models can also not iteratively be improved.

Based on the studies included in the current systematic review, it appears that research on the effect of psychosocial work exposures on mental health complaints of young workers is for the biggest part inspired by the existing, classic occupational health models (i.e., job demand control [resources] model, Effort-Reward Imbalance). To date, research has paid insufficient attention to exposures that are potentially getting more relevant in an increasingly digitalized and intensified work environment–such as interruptions at work, and challenges related to increased standardization and documentation of work, while these exposures might be particularly relevant for young workers’ mental health. Based on the same need for better capturing the contemporary psychosocial work environment, the DYNAMIK questionnaire has been developed, which is explicitly aimed at reflecting modern day work including risk factors such as interruption of work, usability of technology used at work, and work during leisure time (Diebig et al. 2020). An overarching framework integrating existing models and new insights can help guiding research and can facilitate knowledge accumulation. The model suggested by Harvey et al. (2017) as a result of their meta-review might be helpful. Nevertheless, this model is more a framework in the sense that it categorizes concepts, while it does not facilitate deduction of testable hypotheses and it does not articulate interdependencies of psychosocial work factors.

Still, using such a broadly accepted framework does not address another potential issue affecting research and practice, namely that a worker’s mental health is part of a complex system, which includes the workplace. Harvey et al. (2017) conclude in their meta-review that there is no one “toxic factor” underlying mental health complaints. For practice this means that there is not one universally applicable aspect of the psychosocial work environment, i.e. the “toxic factor” that must be fixed in order to improve work-related mental health complaints. For scientific research this implies to reconsider the way research is designed, conducted, and analysed, because occupational mental health research currently follows a reductionist approach in which researchers are trying to identify the most parsimonious unidirectional exposure-outcome relationships, aiming at identifying the most toxic exposures for mental health complaints.

By simply adding more up to date exposures to studies that better reflect contemporary workspaces, complexity is still not taken into account and researchers implicitly keep on looking for the toxic factors ought to explain mental health complaints. Understanding a worker’s health as a complex system implies that a psychosocial work exposure that might not appear in research on one-on-one associations with mental health complaints, could play a crucial role within the actual system by triggering effects that are then manifested by more obvious and bigger changes in other constructs (Fried and Robinaugh 2020). Translating a complexity approach into research practice arguably has a huge potential for the field of occupational mental health research (Olthof et al. 2020).

### Strengths

This systematic review attempts to be the most comprehensive and up to date overview of the effect of psychosocial work factors on mental health complaints of young workers. All search terms were selected with having this particular group in mind and with no time limit, making it an extensive systematic review that covers all relevant mental health outcomes and psychosocial work exposures. The application of the GRADE framework made it more explicit than two previous reviews that the certainty of evidence is generally very low.

### Limitations

By excluding clinical outcomes, it is possible that some informative articles have been missed. Also, studies were excluded if it could not be ruled out that workers older than 35 years were included in the sample, which can be considered a too strict inclusion criterion given that a sample might still be representative for young workers even if it included a few workers older than 35. We could not retrieve five studies, which might have resulted in an uncomplete picture. Finally, the harmonization of outcomes and exposures was not determined a priori. The aim of the harmonization was to enable the synthesis of results. It can be argued that the harmonization choices that were made after data extraction are open to debate and that, for example, psychological job demands and job pressure should not be given the same term, because they are conceptually close but still distinct.

### Implications for future research

Arguably, research on effects of psychosocial work factors on mental health complaints is often guided by which variables happen to be in a questionnaire that mostly serves several other purposes. Instead of testing ill-specified hypotheses on observational data and running confirmatory analyses, more exploratory research has the potential to help shape better hypotheses. These hypotheses can then be answered using more tailored data and in a methodologically sound manner, a priori making the hypothesized causal structure of the assessed exposure-outcome association explicit. Such exploratory research should address the aforementioned challenges of occupational mental health research. This can firstly be achieved by integrating recent developments on mental health complaint classifications and psychosocial work factor frameworks, including contemporary exposures. Secondly, research designs and analyses methods should be able to reflect the features of worker’s mental health as a complex system. This can be facilitated by making more use of longitudinal data and qualitative designs and by applying recently advanced analysis methods that can model complexity (Bringmann et al. 2022).

Across the studies included in the current systematic review, the authors expect systematic differences between younger and older workers concerning which psychosocial work factors affect mental health based on the literature underlying their studies (Akkermans et al. 2009, 2013a, b; Cheng et al. 2013; Haley et al. 2013; Klug 2020; Milner et al. 2017; Salmela-Aro and Upadyaya 2018; Shi et al. 2018; Zoer et al. 2011). Some hypothesizing on unique work-related needs of young workers can be found. It is argued that the changes and increase of responsibility that young workers are facing puts them in more need of job resources (Akkermans et al. 2013a, b; Shi et al. 2018) (e.g., job control) (Milner et al. 2017)) and that perceived job insecurity plays a central role for young workers (Akkermans et al. 2009; Cheng et al. 2013; Klug 2020).

As mentioned above, robust evidence for associations between psychosocial working conditions and mental health complaints can be found for the general working population including workers of all ages. The knowledge on these known associations can systematically be integrated with insights from lifespan research in order to propose work-related vulnerabilities that are particularly relevant for young workers. To give an example, lifespan research suggests that the age in which workers begin their working life is marked by a “shift in motivation from striving for gains to maintenance and prevention of losses” and “change from extrinsic to intrinsic motives for working” (Zacher and Froidevaux 2021). It can consequently be hypothesized that for young workers, low organizational justice, which has shown to be associated with mental health complaints in the general working population, is more problematic when it concerns extrinsic motivational aspects of the job such as the distribution of salary, rather than intrinsic motivational aspects of the job such as the distribution of interesting and challenging tasks.

Using this input, even with a lack of research focussing on young workers, a more informed theoretical inference can be made on how to translate evidence form the general working population including young workers to young workers in particular.

## Conclusion

Work-related ill mental health is a persistent and potentially increasing phenomenon among young workers. The psychosocial quality of the workplace should be created and maintained in such a way that work positive contribution to the mental health of young workers. The certainty of evidence on psychosocial work factors and mental health outcomes was found to be very low, therefore not enough is known on which psychosocial work factors affect the mental health of young workers to give evidence-based guidance to practice. This leaves practitioners with potentially inaccurate or incomplete information for creating healthy work.

## Supplementary Information

Below is the link to the electronic supplementary material.

Supplementary file1 (PDF 149 KB)
