# Supplementary material for: Psychosocial work factors affecting mental health of young workers: a systematic review
Source: Int Arch Occup Environ Health. 2022 Aug 17;96(1):57–75. doi: 10.1007/s00420-022-01907-y (PMC9823059; doi:10.1007/s00420-022-01907-y)
Supplement: Supplementary file 1 — Supplementary file1 (PDF 149 KB) [file 420_2022_1907_MOESM1_ESM.pdf]

Psychosocial work factors affecting mental health of young workers: a systematic review

Van Veen M[1,2,3,5], Oude Hengel KM[1,3], Schelvis, RMC[3,4,5], Bongers PM[1,2,3], Ket JCF[6], van der Beek AJ[2,3,5], Boot CRL[2,3,5]

[1] Netherlands Organisation for Applied Scientific Research TNO, Unit Healthy Living, Leiden, The Netherlands

[2] Amsterdam UMC location Vrije Universiteit Amsterdam, Public and Occupational Health, Boelelaan 1117, Amsterdam, The Netherlands

[3] Body@Work, Research Center on Work, Health and Technology, TNO/VUmc, Amsterdam, The Netherlands

[4] Amsterdam UMC location University of Amsterdam, Public and Occupational Health, Meibergdreef 9, Amsterdam, The Netherlands

[5] Amsterdam Public Health, Societal Participation & Health, Amsterdam, The Netherlands

[6] Medical Library, Vrije Universiteit Amsterdam, Amsterdam, The Netherlands,

Supplementary File 1 – Full Search Strategy and Prisma Checklist

Full Search Strategy

Search strategy for PubMed (7 October 2021)

Search	Query	Results
#6	#1 AND #2 AND #3 AND #4 AND #5	4,835
#5	"Personal Autonomy"[Mesh] OR "Professional Autonomy"[Mesh] OR "Workload"[Mesh] OR "Recreation"[Mesh:NoExp] OR "Reward"[Mesh:NoExp] OR "amount of work"[tiab] OR "choice overload*"[tiab] OR "cognitive demand*"[tiab] OR "collegial support"[tiab] OR "computer anger*"[tiab] OR "computer rag*"[tiab] OR "co-worker support*"[tiab] OR "decision authorit*"[tiab] OR "decisional latitude*"[tiab] OR "device related anger*"[tiab] OR "device related anxiet*"[tiab] OR "emotional demand*"[tiab] OR "fear of better option*"[tiab] OR "fear of missing out"[tiab] OR "job demand*"[tiab] OR "job insecurit*"[tiab] OR "job securit*"[tiab] OR "managerial support*"[tiab] OR "mental load*"[tiab] OR "mental work load*"[tiab] OR "mental workload*"[tiab] OR "organisational climate*"[tiab] OR "organisational culture*"[tiab] OR "organizational climate*"[tiab] OR "organizational culture*"[tiab] OR "over choice"[tiab] OR "personal develop*"[tiab] OR "personal grow*"[tiab] OR "procedural fairness"[tiab] OR "procedural justice"[tiab] OR "psychological safet*"[tiab] OR "quality demand*"[tiab] OR "role ambiguit*"[tiab] OR "role uncertaint*"[tiab] OR "role unclarit*"[tiab] OR "social cohesi*"[tiab] OR "social comparison*"[tiab] OR "social support"[tiab] OR "supervisor support*"[tiab] OR "support by colleag*"[tiab] OR "support by the manager*"[tiab] OR "task difficult*"[tiab] OR "time pressure*"[tiab] OR "unclear task*"[tiab] OR "work load*"[tiab] OR "workload*"[tiab] OR appreciati*[tiab] OR autonom*[tiab] OR interruption*[tiab] OR overchoice[tiab] OR participation*[tiab] OR recover*[tiab] OR recreati*[tiab] OR relax*[tiab] OR reorganisation*[tiab] OR reorganization*[tiab] OR reward*[tiab] OR technostress*[tiab]	1,494,131

**#4**	"Mental Fatigue"[Mesh] OR "Stress, Psychological"[Mesh] OR "Mental Health"[Mesh] OR "Quality of Life"[Mesh] OR burnout*[tiab] OR "burn out*"[tiab] OR anxi*[tiab] OR depress*[tiab] OR stress*[tiab] OR "mental health"[tiab] OR "quality of life"[tiab] OR "psychological health"[tiab] OR exhaust*[tiab] OR "psychological fatigue*"[tiab] OR "mental fatigue*"[tiab]	**2,012,271**
**#3**	"Epidemiologic Studies"[Mesh] OR cohort[tiab] OR (case[tiab] AND (control[tiab] OR controll*[tiab] OR comparison[tiab] OR referent[tiab])) OR risk[tiab] OR causation[tiab] OR causal[tiab] OR "odds ratio"[tiab] OR etiol*[tiab] OR aetiol*[tiab] OR "natural history"[tiab] OR predict*[tiab] OR prognos*[tiab] OR outcome[tiab] OR course[tiab] OR retrospect*[tiab]	**7,399,545**
**#2**	"Work"[Mesh] OR "Employment"[Mesh:NoExp] OR "Workplace"[Mesh] OR work*[tiab] OR occupation*[tiab] OR profession*[tiab] OR job[tiab] OR jobs[tiab] OR employ*[tiab] OR labor[tiab] OR laborer*[tiab] OR labour*[tiab] OR vocation*[tiab]	**2,763,377**
**#1**	"Young Adult"[Mesh] OR young*[tiab] OR "early career*"[tiab] OR "job starter*"[tiab] OR "short work experience"[tiab]	**1,581,767**

Search strategy for Clarivate Analytics/Web of Science Core Collection (7 October 2021)

Set	Query	Results
**#7**	#5 AND #4 AND #3 AND #2 AND #1	**2819**
**#5**	TS=("amount of work" OR "choice overload*" OR "cognitive demand*" OR "collegial support" OR "computer anger*" OR "computer rag*" OR "co-worker support*" OR "decision authorit*" OR "decisional latitude*" OR "device related anger*" OR "device related anxiet*" OR "emotional demand*" OR "fear of better option*" OR "fear of missing out" OR "job demand*" OR "job insecurit*" OR "job securit*" OR "managerial support*" OR "mental load*" OR "mental work load*" OR "mental workload*" OR "organisational climate*" OR "organisational culture*" OR "organizational climate*" OR "organizational culture*" OR "over choice" OR "personal develop*" OR "personal grow*" OR "procedural fairness" OR "procedural justice" OR "psychological safet*" OR "quality demand*" OR "role ambiguit*" OR "role uncertaint*" OR "role unclarit*" OR "social cohesi*" OR "social comparison*" OR "social support" OR "supervisor support*" OR "support by colleag*" OR "support by the manager*" OR "task difficult*" OR "time pressure*" OR "unclear task*" OR "work load*" OR "workload*" OR "appreciati*" OR "autonom*" OR "interruption*" OR "overchoice" OR "participation*" OR "recover*" OR "recreati*" OR "relax*" OR "reorganisation*" OR "reorganization*" OR "reward*" OR "technostress*")	**2641496**
**#4**	TS=("burnout*" OR "burn out*" OR "anxi*" OR "depress*" OR "stress*" OR "mental health" OR "quality of life" OR "psychological health" OR "exhaust*" OR "psychological fatigue*" OR "mental fatigue*")	**3363595**
**#3**	TS=("cohort" OR ("case" NEAR/3 ("control" OR "controll*" OR "comparison" OR "referent")) OR "risk" OR "causation" OR "causal" OR "odds ratio" OR "etiol*" OR "aetiol*" OR "natural history" OR "predict*" OR "prognos*" OR "outcome" OR "course" OR "retrospect*")	**8638122**
**#2**	TS=("work*" OR "occupation*" OR "profession*" OR "job" OR "jobs" OR "employ*" OR "labor" OR "laborer*" OR "labour*" OR "vocation*")	**5955900**
**#1**	TS=("young*" OR "early career*" OR "job starter*" OR "short work experience")	**1046947**

Search strategy for Ebsco/APA PsycINFO (7 October 2021)

#	Query	Limiters / Expanders	Results
**S8**	S7 AND S3 AND S4 AND S5 AND S6		**5,725**

**S7**	S1 OR S2		**573,704**
**S6**	DE "Autonomy" OR DE "Work Scheduling" OR DE "Division of Labor" OR DE "Recreation" OR DE "Social Support" OR DE "Computer Anxiety" OR DE "Decision Making" OR DE "Job Security" OR DE "Job Satisfaction" OR DE "Supervisor Employee Interaction" OR DE "Organizational Crises" OR DE "Organizational Characteristics" OR DE "Organizational Change" OR DE "Organizational Climate" OR DE "Organizational Structure" OR DE "Decentralization" OR DE "Organizational Behavior" OR DE "Employee Interaction" OR DE "Organizational Citizenship Behavior" OR DE "Organizational Effectiveness" OR DE "Organizational Politics" OR DE "Professional Development" OR DE "Professional Competence" OR DE "Professionalism" OR DE "Professional Recognition" OR DE "Professional Socialization" OR DE "Professional Networking" OR DE "Professional Specialization" OR DE "Occupational Safety" OR DE "Social Networks" OR DE "Online Social Networks" OR DE "Social Support" OR DE "Rewards" OR TI("amount of work" OR "choice overload*" OR "cognitive demand*" OR "collegial support" OR "computer anger*" OR "computer rag*" OR "co-worker support*" OR "decision authorit*" OR "decisional latitude*" OR "device related anger*" OR "device related anxiet*" OR "emotional demand*" OR "fear of better option*" OR "fear of missing out" OR "job demand*" OR "job insecurit*" OR "job securit*" OR "managerial support*" OR "mental load*" OR "mental work load*" OR "mental workload*" OR "organisational climate*" OR "organisational culture*" OR "organizational climate*" OR "organizational culture*" OR "over choice" OR "personal develop*" OR "personal grow*" OR "procedural fairness" OR "procedural justice" OR "psychological safet*" OR "quality demand*" OR "role ambiguit*" OR "role uncertaint*" OR "role unclarit*" OR "social cohesi*" OR "social comparison*" OR "social support" OR "supervisor support*" OR "support by colleag*" OR "support by the manager*" OR "task difficult*" OR "time pressure*" OR "unclear task*" OR "work load*" OR "workload*" OR appreciati* OR autonom* OR interruption* OR overchoice OR participation* OR recover* OR recreati* OR relax* OR reorganisation* OR reorganization* OR reward* OR technostress*) OR AB("amount of work" OR "choice overload*" OR "cognitive demand*" OR "collegial support" OR "computer anger*" OR "computer rag*" OR "co-worker support*" OR "decision authorit*" OR "decisional latitude*" OR "device related anger*" OR "device related anxiet*" OR "emotional demand*" OR "fear of better option*" OR "fear of missing out" OR "job demand*" OR "job insecurit*" OR "job securit*" OR "managerial support*" OR "mental load*" OR "mental work load*" OR "mental workload*" OR "organisational climate*" OR "organisational culture*" OR "organizational climate*" OR "organizational culture*" OR "over choice" OR "personal develop*" OR "personal grow*" OR "procedural fairness" OR "procedural justice" OR "psychological safet*" OR "quality demand*" OR "role ambiguit*" OR "role uncertaint*" OR "role unclarit*" OR "social cohesi*" OR "social comparison*" OR "social support" OR "supervisor support*" OR "support by colleag*" OR "support by the manager*" OR "task difficult*" OR "time pressure*" OR "unclear task*" OR "work load*" OR		**95,551**

	"workload*" OR appreciati* OR autonom* OR interruption* OR overchoice OR participation* OR recover* OR recreati* OR relax* OR reorganisation* OR reorganization* OR reward* OR technostress*) OR KW("amount of work" OR "choice overload*" OR "cognitive demand*" OR "collegial support" OR "computer anger*" OR "computer rag*" OR "co-worker support*" OR "decision authorit*" OR "decisional latitude*" OR "device related anger*" OR "device related anxiet*" OR "emotional demand*" OR "fear of better option*" OR "fear of missing out" OR "job demand*" OR "job insecurit*" OR "job securit*" OR "managerial support*" OR "mental load*" OR "mental work load*" OR "mental workload*" OR "organisational climate*" OR "organisational culture*" OR "organizational climate*" OR "organizational culture*" OR "over choice" OR "personal develop*" OR "personal grow*" OR "procedural fairness" OR "procedural justice" OR "psychological safet*" OR "quality demand*" OR "role ambiguit*" OR "role uncertaint*" OR "role unclarit*" OR "social cohesi*" OR "social comparison*" OR "social support" OR "supervisor support*" OR "support by colleag*" OR "support by the manager*" OR "task difficult*" OR "time pressure*" OR "unclear task*" OR "work load*" OR "workload*" OR appreciati* OR autonom* OR interruption* OR overchoice OR participation* OR recover* OR recreati* OR relax* OR reorganisation* OR reorganization* OR reward* OR technostress*)		
**S5**	DE "Fatigue" OR DE "Stress" OR DE "Academic Stress" OR DE "Chronic Stress" OR DE "Environmental Stress" OR DE "Financial Strain" OR DE "Occupational Stress" OR DE "Physiological Stress" OR DE "Posttraumatic Stress" OR DE "Psychological Stress" OR DE "Social Stress" OR DE "Stress Reactions" OR DE "Distress" OR DE "Mental Health" OR DE "Mental Status" OR DE "Quality of Life" OR DE "Health Related Quality of Life" OR DE "Quality of Work Life" OR DE "Quality of Life Measures" OR DE "Anxiety" OR DE "Anxiety Sensitivity" OR DE "Performance Anxiety" OR DE "Social Anxiety" OR DE "Depression (Emotion)" OR TI(burnout* OR "burn out*" OR anxi* OR depress* OR stress* OR "mental health" OR "quality of life" OR "psychological health" OR exhaust* OR "psychological fatigue*" OR "mental fatigue*") OR AB(burnout* OR "burn out*" OR anxi* OR depress* OR stress* OR "mental health" OR "quality of life" OR "psychological health" OR exhaust* OR "psychological fatigue*" OR "mental fatigue*") OR KW(burnout* OR "burn out*" OR anxi* OR depress* OR stress* OR "mental health" OR "quality of life" OR "psychological health" OR exhaust* OR "psychological fatigue*" OR "mental fatigue*")		**146,592**
**S4**	DE "Epidemiology" OR DE "Cohort Analysis" OR DE "Causal Analysis" OR DE "Causality" OR DE "Etiology" OR DE "Prognosis" OR DE "Longitudinal Studies" OR DE "Prospective Studies" OR TI(cohort OR (case N3 (control OR controll* OR comparison OR referent)) OR risk OR causation OR causal OR "odds ratio" OR etiol* OR aetiol* OR "natural history" OR predict* OR prognos* OR outcome OR course OR retrospect*) OR AB(cohort OR (case N3 (control OR controll* OR comparison OR referent)) OR risk OR causation OR causal OR "odds ratio" OR etiol* OR aetiol* OR "natural history" OR predict* OR prognos* OR outcome OR course OR retrospect*) OR KW(cohort OR (case N3 (control OR controll* OR		**253,303**

	comparison OR referent)) OR risk OR causation OR causal OR "odds ratio" OR etiol* OR aetiol* OR "natural history" OR predict* OR prognos* OR outcome OR course OR retrospect*)		
**S3**	DE "Employment Status" OR DE "Occupations" OR DE "Reemployment" OR DE "Child Care Workers" OR DE "Emergency Personnel" OR DE "Fire Fighters" OR DE "First Responders" OR DE "Paramedics" OR DE "Police Personnel" OR DE "Rescue Workers" OR DE "Social Workers" OR DE "Professional Personnel" OR DE "Personnel" OR DE "Anthropologists" OR DE "Clinicians" OR DE "Counselors" OR DE "Counselor Characteristics" OR DE "Counselor Trainees" OR DE "Rehabilitation Counselors" OR DE "School Counselors" OR DE "Vocational Counselors" OR DE "Educational Personnel" OR DE "School Administrators" OR DE "School Counselors" OR DE "School Nurses" OR DE "Teacher Aides" OR DE "Teachers" OR DE "Health Personnel" OR DE "Allied Health Personnel" OR DE "Caregivers" OR DE "Medical Personnel" OR DE "Mental Health Personnel" OR DE "Information Specialists" OR DE "Librarians" OR DE "Legal Personnel" OR DE "Attorneys" OR DE "Judges" OR DE "Law Enforcement Personnel" OR DE "Physicists" OR DE "Psychologists" OR DE "Clinical Psychologists" OR DE "Counseling Psychologists" OR DE "Educational Psychologists" OR DE "Experimental Psychologists" OR DE "Industrial Psychologists" OR DE "Military Psychologists" OR DE "Social Psychologists" OR DE "Scientists" OR DE "Sociologists" OR DE "Therapists" OR DE "Occupational Therapists" OR DE "Physical Therapists" OR DE "Psychotherapists" OR DE "Speech Therapists" OR DE "Therapist Trainees" OR DE "Working Conditions" OR DE "Occupational Safety" OR DE "Working Space" OR TI(work* OR occupation* OR profession* OR job OR jobs OR employ* OR labor OR laborer* OR labour* OR vocation*) OR AB(work* OR occupation* OR profession* OR job OR jobs OR employ* OR labor OR laborer* OR labour* OR vocation*) OR KW(work* OR occupation* OR profession* OR job OR jobs OR employ* OR labor OR laborer* OR labour* OR vocation*)		**140,203**
**S2**	TI(young* OR "early career*" OR "job starter*" OR "short work experience") OR AB(young* OR "early career*" OR "job starter*" OR "short work experience") OR KW(young* OR "early career*" OR "job starter*" OR "short work experience")		**76,548**
**S1**		Limiters - Age Groups: Young Adulthood (18-29 yrs)	**573,704**

PRISMA 2020 Checklist

Section and Topic	Item #	Checklist item	Location where item is reported
**TITLE**			
Title	1	Identify the report as a systematic review.	Title
**ABSTRACT**			
Abstract	2	See the PRISMA 2020 for Abstracts checklist.	Abstract
**INTRODUCTION**			
Rationale	3	Describe the rationale for the review in the context of existing knowledge.	Introduction
Objectives	4	Provide an explicit statement of the objective(s) or question(s) the review addresses.	Introduction
**METHODS**			
Eligibility criteria	5	Specify the inclusion and exclusion criteria for the review and how studies were grouped for the syntheses.	Methods
Information sources	6	Specify all databases, registers, websites, organisations, reference lists and other sources searched or consulted to identify studies. Specify the date when each source was last searched or consulted.	Methods
Search strategy	7	Present the full search strategies for all databases, registers and websites, including any filters and limits used.	Supplementary file
Selection process	8	Specify the methods used to decide whether a study met the inclusion criteria of the review, including how many reviewers screened each record and each report retrieved, whether they worked independently, and if applicable, details of automation tools used in the process.	Methods
Data collection process	9	Specify the methods used to collect data from reports, including how many reviewers collected data from each report, whether they worked independently, any processes for obtaining or confirming data from study investigators, and if applicable, details of automation tools used in the process.	Methods
Data items	10a	List and define all outcomes for which data were sought. Specify whether all results that were compatible with each outcome domain in each study were sought (e.g. for all measures, time points, analyses), and if not, the methods used to decide which results to collect.	Methods
	10b	List and define all other variables for which data were sought (e.g. participant and intervention characteristics, funding sources). Describe any assumptions made about any missing or unclear information.	Methods
Study risk of bias assessment	11	Specify the methods used to assess risk of bias in the included studies, including details of the tool(s) used, how many reviewers assessed each study and whether they worked independently, and if applicable, details of automation tools used in the process.	Methods
Effect measures	12	Specify for each outcome the effect measure(s) (e.g. risk ratio, mean difference) used in the synthesis or presentation of results.	n.a.
Synthesis methods	13a	Describe the processes used to decide which studies were eligible for each synthesis (e.g. tabulating the study intervention characteristics and comparing against the planned groups for each synthesis (item #5)).	Methods
	13b	Describe any methods required to prepare the data for presentation or synthesis, such as handling of missing summary statistics, or data conversions.	n.a.
	13c	Describe any methods used to tabulate or visually display results of individual studies and syntheses.	n.a.
	13d	Describe any methods used to synthesize results and provide a rationale for the choice(s). If meta-analysis was performed, describe the model(s), method(s) to identify the presence and extent of statistical heterogeneity, and software package(s) used.	Methods
	13e	Describe any methods used to explore possible causes of heterogeneity among study results (e.g. subgroup analysis, meta-regression).	n.a.
	13f	Describe any sensitivity analyses conducted to assess robustness of the synthesized results.	n.a.
Reporting bias assessment	14	Describe any methods used to assess risk of bias due to missing results in a synthesis (arising from reporting biases).	Results

PRISMA 2020 Checklist

Section and Topic	Item #	Checklist item	Location where item is reported
Certainty assessment	15	Describe any methods used to assess certainty (or confidence) in the body of evidence for an outcome.	Methods
**RESULTS**			
Study selection	16a	Describe the results of the search and selection process, from the number of records identified in the search to the number of studies included in the review, ideally using a flow diagram.	Results
	16b	Cite studies that might appear to meet the inclusion criteria, but which were excluded, and explain why they were excluded.	Not reported
Study characteristics	17	Cite each included study and present its characteristics.	Table 1
Risk of bias in studies	18	Present assessments of risk of bias for each included study.	Table 2
Results of individual studies	19	For all outcomes, present, for each study: (a) summary statistics for each group (where appropriate) and (b) an effect estimate and its precision (e.g. confidence/credible interval), ideally using structured tables or plots.	Table 3
Results of syntheses	20a	For each synthesis, briefly summarise the characteristics and risk of bias among contributing studies.	Table 4
	20b	Present results of all statistical syntheses conducted. If meta-analysis was done, present for each the summary estimate and its precision (e.g. confidence/credible interval) and measures of statistical heterogeneity. If comparing groups, describe the direction of the effect.	n.a.
	20c	Present results of all investigations of possible causes of heterogeneity among study results.	n.a.
	20d	Present results of all sensitivity analyses conducted to assess the robustness of the synthesized results.	n.a.
Reporting biases	21	Present assessments of risk of bias due to missing results (arising from reporting biases) for each synthesis assessed.	Results
Certainty of evidence	22	Present assessments of certainty (or confidence) in the body of evidence for each outcome assessed.	Table 4
**DISCUSSION**			
Discussion	23a	Provide a general interpretation of the results in the context of other evidence.	Discussion
	23b	Discuss any limitations of the evidence included in the review.	Discussion
	23c	Discuss any limitations of the review processes used.	Discussion
	23d	Discuss implications of the results for practice, policy, and future research.	Discussion
**OTHER INFORMATION**			
Registration and protocol	24a	Provide registration information for the review, including register name and registration number, or state that the review was not registered.	Introduction
	24b	Indicate where the review protocol can be accessed, or state that a protocol was not prepared.	Methods
	24c	Describe and explain any amendments to information provided at registration or in the protocol.	n.a.
Support	25	Describe sources of financial or non-financial support for the review, and the role of the funders or sponsors in the review.	Abstract
Competing interests	26	Declare any competing interests of review authors.	Discussion
Availability of data, code and other materials	27	Report which of the following are publicly available and where they can be found: template data collection forms; data extracted from included studies; data used for all analyses; analytic code; any other materials used in the review.	n.a.

From: Page MJ, McKenzie JE, Bossuyt PM, Boutron I, Hoffmann TC, Mulrow CD, et al. The PRISMA 2020 statement: an updated guideline for reporting systematic reviews. BMJ 2021;372:n71. doi: 10.1136/bmj.n71
